# PROTOCOL: Testing frequency and student achievement: A systematic review

**DOI:** 10.1002/cl2.1212

**Published:** 2022-01-07

**Authors:** Morten K. Thomsen, Julie K. Seerup, Jens Dietrichson, Anja Bondebjerg, Bjørn C. A. Viinholt

**Affiliations:** ^1^ Department of Sociology University of Oxford Oxford UK; ^2^ VIVE—The Danish Center for Social Science Research Copenhagen Denmark

## Abstract

This is the protocol for a Campbell review. Our primary research question is: What are the effects of different testing frequencies on student achievement? Our secondary research question is: What are the effects of different testing frequencies on measures of students' testing anxiety? Our third research question is: How are the effects of different testing frequencies on student achievement and testing anxiety moderated by subject, grade, type of test, duration of the intervention, and gender?

## 1 BACKGROUND

### 1.1 Description of the condition

School‐based testing is often well suited and widely used for monitoring students' academic progress. However, for almost a century, researchers have taken an interest in the potential moderating qualities of testing student's academic achievements *on their academic achievements* (Jones, 1923; Keys, 1934; Kulp, 1933; Spitzer, 1939; Turney, 1931); that is, how does exposing students to school‐based testing at different frequencies contribute to their academic progress?

In spite of many attempts to answer the above question and to formulate guidelines for how to use school‐based testing for the benefit of students, educational scholars, policy makers, and teachers are yet to fully understand the potential effects of school‐based testing on the academic achievement of students (e.g., Bergbauer et al., 2018; National Research Council [NRC], 2011; World Bank, 2017).

In the latter years, tests have become associated with the accountability of national educational systems and are now widely used in international comparisons of student achievement. Since the 1970s, scholars have debated this phenomenon, sometimes referred to as ‘the global testing culture’ or ‘the educational reform movement’ (NRC, 2011; Sahlberg, 2010; Smith, 2016). This trend encompasses an increased political focus on accountability and assessment of learning in educational systems and has fostered both national and international testing schemes, such as the Programme for International Student Assessment (PISA), the Progress in International Reading Literacy Study (PIRLS), and the Trends In Mathematics and Science Study (TIMMS). Furthermore, various national educational policies involving an increased focused on rigorous testing, such as the No Child Left Behind Act in the United States, have been implemented.

The testing materials in these aforementioned testing schemes and national policies typically consist of standardised tests, which allow comparisons between separate school systems, for example across districts or countries. With the rapid development of new (computer) technologies, the practicalities of testing students become easier to manage. Some would argue that testing students is an inevitable tool in present day educational systems to secure and demonstrate accountability and guide teachers and managers to create optimal learning environments for students (Buck et al., 2010; Crooks, 1988; NRC, 2011). However, there is an ongoing policy debate on the impact of testing on students' academic achievement (e.g., Bergbauer et al., 2018; NRC, 2011). Testing has acquired a negative connotation for many practitioners, particularly when it comes to standardised high‐stakes, summative testing (Rawson & Dunlosky, 2012). Opponents of standardised testing schemes often refer to adverse consequences such as teachers focusing on ‘teaching to the test’, and that frequent testing fosters distress and emotional difficulties among students (DordiNejad et al., 2011; Organization for Economic Co‐operation and Development [OECD], 2017).

Testing takes time away from other modes of instruction (Rawson & Dunlovsky, 2012). Also, implementing large‐scale standardised testing, like in the No Child Left Behind policy, is expensive (Azin & Resendez, 2008). It is therefore important to examine the effects of more frequent testing on student achievement. Furthermore, as there may be adverse effects of testing students more frequently, decisions on the testing frequency should not only consider achievement but also the impact on students' emotional well‐being. The aim of this systematic review is to examine how changes in the frequency of school‐based tests affect academic achievement (primary outcome measure) and testing‐related anxiety (secondary outcome measure) in primary and secondary school students.

The effects of changing the testing frequency may depend on the subject students are tested in, how the tests are conducted, and on student characteristics. Feedback from school‐based tests may be more important in some subjects than others (Azmat & Irriberri, 2010), and testing anxiety often differs across subjects (e.g., Wolters & Pintrich, 1998). The age and grade of students may moderate effect sizes (Adesope et al., 2017), for example, because the grade affects how important test information is for teachers, and students react differently to being tested at different ages. As evidenced by the critique against standardised and high‐stakes testing, the type of test may matter for both achievement and anxiety. Higher exposure to an increased testing frequency may affect students differently compared to less intensive exposure (Yang et al., 2021). Earlier findings indicate that females are more likely to experience and report higher levels of testing anxiety than males (DordiNejad et al., 2011; OECD, 2017; Segool et al., 2013; von der Embse et al., 2017). We will examine potential heterogeneity using subject, grade level, type of test, the duration of the intervention, and gender as moderators.

### 1.2 Description of the intervention

The aim of this systematic review is to examine the potential effects of testing frequency alterations on the academic achievement of primary and secondary school students. Whereas specific criteria for exclusion, including those concerning the intervention, are thoroughly described in Section 3, this section serves as a description of the interventions of potential interest. We have six criteria that any intervention must meet to be of relevance to this systematic review:

1. Interventions must alter the testing frequency for at least one student group and provide information about the benchmark testing frequency.
2. To secure the ecological validity of results, interventions must be implemented within a school setting. Hence, laboratory‐based interventions are not included.
3. Interventions must be aimed at either primary or secondary school students. Interventions set in tertiary education, such as university settings, or in pre‐school settings are not included.
4. To strengthen construct validity, interventions should only manipulate the testing frequency, and not incorporate additional components. As a result, interventions combining changes in testing frequency (e.g., by introducing curriculum‐based measurement) with other components (e.g., peer‐assisted learning strategies) are not eligible for inclusion.
5. Applied tests must be recorded on paper, computer, tablet, and so forth. Orally performed tests are included if their results are recorded.
6. The effects of the interventions should not be evaluated by tests that are identical to the ones used during the intervention. If the post‐intervention tests are identical to the tests used during the intervention, chances are that students might learn the specific questions and answers by heart, thus introducing a confounding element. Otherwise, there is no restriction on the type of test used during the intervention, or to evaluate the effects of the intervention. Both standardised and non‐standardised tests as well as formative and summative tests, and high‐stakes and low‐stakes exams will be included.

### 1.3 How the intervention might work

Theoretically, testing students may have both beneficial and adverse effects on achievement and anxiety.

In favour of increased testing frequencies, numerous researchers have suggested that students achieve more when frequent testing is implemented. Test results may provide teachers with information about a given student's difficulties and strengths, thus serving to qualify personal feedback (Dunlosky et al., 2013; McDaniel et al., 2007; Rawson & Dunlosky, 2012) and allowing for better aimed individual and class‐level instruction (Black & Wiliam, 2009). Tests may imply that students receive feedback (with or without teacher mediation) that allows them to correct errors (Adesope et al., 2017). Furthermore, tests might act as extrinsic motivators, leading students to study harder (Bernatzsky, Cabrera, & Cid, 2017), and teachers and schools to increase efforts to improve student achievement (Woessmann, 2002). In addition, it has been suggested that frequent testing administered in the form of practice tests (formative tests) has the potential to improve student retention and thereby learning (Carpenter et al., 2009; Carpenter, 2012; Dunlosky et al., 2013; Glover, 1989; Karpicke & Aue, 2015; Rawson & Dunlosky, 2012; Rowland, 2014; Yang et al., 2021).

The phenomenon that tests may improve learning through improved retention is often called the ‘testing effect’, or ‘test‐enhanced learning’ (Yang et al., 2021). In the testing literature, two main hypotheses exist regarding how the testing effect comes into existence. The first hypothesis, ‘amount‐of‐processing’, states that exposure to the material is vital for recall: the more exposure, the more probable a correct recall will become. In this perspective, testing is seen as an additional exposure to the material. The second hypothesis, which focuses on the retrieval process of recall, states that retrieving an item from memory will strengthen the retrieval route or create new retrieval routes, which then increases the chance of correct recall afterwards (Dempster, 1996). The latter hypothesis has gained increased attention since research indicates that not only exposure to the material, but rather the act of processing and retrieving itself strengthens recall. Thus, active repetition of the material, for example, taking a recall test, has been shown to yield greater long‐term retention of a material than passive repetition, such as re‐studying (Roediger III & Karpicke, 2006a, 2006b).

Previous findings support the common sense assumption that increasing the testing frequency indefinitely does not result in indefinite increases in student achievement (Bangert‐Drowns et al., 1991). In other words, doing 10 tests is not necessarily ten times as good as doing one; on the contrary, 'over‐testing' may have the potential to lead to decreases in student achievement. One reason for this is that testing is likely to take time away from instruction and at some point, more instruction time will be more valuable for student achievement (Rawson & Dunlovsky, 2012). Strengthened extrinsic motivation may lead to unwanted focus on the tested material (i.e., ‘teaching to the test’) or more generally on tasks that are tested, which is an extra pertinent risk as schools are 'multi‐tasking' organisations (Holmström & Milgrom, 1991). Being exposed to testing is associated with a certain amount of stress and potential demotivation (Cheek et al., 2002). Furthermore, frequent testing might lead to higher levels of testing anxiety.

Testing anxiety occurs in situations where one's skills are being evaluated and is most commonly defined in the literature as ‘a set of phenomenological, physiological, and behavioural responses that accompany concern about the possible negative consequences of failure on an exam’ (Zeidner, 1998, p. 17). High levels of testing anxiety can have adverse consequences for students' academic achievement and may have a negative impact on general life satisfaction (OECD, 2017; von der Embse & Hasson, 2012; von der Embse et al., 2017). Although testing anxiety is a highly relevant phenomenon to consider when reviewing the effects of tests on students' academic achievement, evidence on whether or not an increased testing frequency actually influences students' anxiety levels remains unclear. According to PISA, there is no significant relationship between the frequency of tests (both standardised and teacher‐developed) and reported levels of testing anxiety (OECD, 2017). It is conceivable that the testing frequency has a nonlinear effect on anxiety. For example, going from zero to one test may increase anxiety, whereas increasing the frequency further may familiarise students with conducting tests and thereby decrease anxiety.

As indicated briefly in Section 1.1, changing the testing frequency may have heterogeneous effects on both achievement and anxiety. We will examine whether variables related to subject, student, and test characteristics moderate effect sizes, and we motivate these analyses here.

Feedback from school‐based tests may be more important in some subjects than others, which is one potential explanation of the heterogeneous effects of performance feedback (Azmat & Iriberri, 2010). Feedback may for example be more pertinent in subjects that students are less likely to receive performance information about from other sources than schools and teachers, and students may react differently to feedback depending on the perceived importance of the subject. As feedback is one reason why testing may affect achievement, these explanations may also be relevant for changes of the testing frequency. Furthermore, students self‐reported levels of testing anxiety differ across subjects (Wolters & Pintrich, 1998), which may directly affect how the testing frequency affects measures of testing anxiety. Differential anxiety across subjects may indirectly also make the effects on student achievement heterogeneous, if anxiety in turn affects achievement (von der Embse & Hasson, 2012; von der Embse et al., 2017). We therefore intend to examine heterogeneity of effect sizes across subjects.

Information from tests may be more important when teachers know less about their students, and when students know less about their own performance (Azmat & Iriberri, 2010). For example, both teachers and students may know less about students' achievement level because students have just started school, and teachers may know less because students have transitioned between teachers or because it is harder to keep track of individual students in later grades as classes are larger. Students' emotional reactions to being tested may also depend on their age, and how used they are to test‐taking. We therefore believe it is important to examine the students' grade as a potential source of heterogeneity.

The type of test may matter for both achievement and anxiety. High‐stakes tests have more potential to strengthen extrinsic motivation, and therefore to amplify both beneficial and harmful effects of such motivation. Some studies show that students report significantly higher anxiety levels on standardised high‐stakes (summative) tests versus classroom (formative) tests (Segool et al., 2013). Yet others argue that shorter, more frequent practice tests yield more positive outcomes for learning and life satisfaction than longer, infrequent summative tests (Dunlosky et al., 2013; Rawson & Dunlosky, 2012).

Higher exposure to an increased testing frequency may affect students differently than lower exposure (Yang et al., 2021). Exposure or dosage may have several aspects, for example in terms of the duration of the intervention (e.g., Dietrichson et al., 2021), or in the number of subjects the new testing regime encompasses (Yang et al., 2021). We will focus on the duration of the intervention in our moderator analysis.

Finally, earlier findings indicate that females are more likely to experience and report higher levels of testing anxiety than males (DordiNejad et al., 2011; OECD, 2017; Segool et al., 2013; von der Embse et al., 2017). In this regard, PISA has found a large gender imbalance of 17 percentage points, with 64% of girls compared to 47% of boys reported to be ‘very anxious before a test, even when they are well prepared’ (OECD, 2017). There is reason to suspect that males and females might react differently to alterations in testing frequency, and we plan to include gender as a moderator in our statistical analyses.

### 1.4 Why it is important to do this review

A review similar to this review was published in 1991 by Bangert‐Drowns, Kulik and Kulik. They found an average effect that significantly favoured the experimental groups, that is, the groups that received more frequent testing. Along with these results, the authors also concluded that ‘differences in the higher end of the spectrum might be unimportant for student learning’ (Bangert‐Drowns et al., 1991). Nonetheless, it is not evident from the review what testing frequencies constitute the higher end of the spectrum.

In addition to the considerably longer and up to date search period, there are two main differences, which set our review apart from that of Bangert‐Drowns et al. (1991). Firstly, Bangert‐Drowns et al. (1991) focus on students attending secondary school or higher education. This population is different from ours in that we include children in primary school, but exclude students in tertiary education. Thus, only the secondary school category overlaps between the two reviews. Second, Bangert‐Drowns et al. (1991) limited their search to include only studies from the US, whereas we will make no restrictions based on geography.

A recent review by Yang et al. (2021) examined if testing increased learning from elementary school to university and continuing education. They included only studies in which instruction took place in a classroom but excluded all studies in which control groups also received some test. Their effect sizes thus concern the contrast between more tests and no tests. Our review will include also studies that contrast two groups with any differences in testing frequencies. Further differences between our review and Yang et al. (2021) are that we will examine the impact on testing anxiety, conduct a risk of bias assessment, include studies written in other languages than English, and that we will exclude studies conducted in tertiary education settings.

Two other related reviews are Adesope et al. (2017) and Phelps (2012). The review by Adesope et al. (2017) focused on the effect of low‐stakes practice tests (which are close to our definition of formative tests). Our review also includes summative tests, such as high‐stakes standardised tests, as the potential effect of the increased use of such tests is interesting in a policy perspective. Phelps (2012) included both high‐stakes and low‐stakes tests, but otherwise presented no clearly formulated inclusion criteria in the review article. Adesope et al. (2017) reported effect sizes for ‘one’ and ‘two or more’ practice tests compared to none. Phelps (2012) reported one effect size where the treatment group is ‘tested more frequently than the control group'. Thus, none of the two reviews fully answered our primary research question. As neither Adesope et al. (2017) nor Phelps (2012) reported analyses of testing anxiety, they did not answer our second research question either.

In relation to this present review, further differences are that neither Adesope et al. (2017) nor Phelps (2012) reported a risk of bias assessment of the included studies in their reviews. Both reviews only performed one‐by‐one moderator analyses, which leave several questions unanswered (i.e., the effect size of interventions in primary school is not reported by either review). A large share of studies in Adesope et al. (2017) used identical practice and outcome tests, which may capture only rote learning and is a study design we will exclude. Most studies in Adesope et al. (2017) were laboratory experiments, which we also do not intend to include in the present review.

Other researchers who reviewed related topics concerning test‐enhanced learning are Black and Wiliam (2009), Fuchs and Fuchs (2001), Karpicke and Grimaldi (2012), Kingston and Nash (2011), McDaniel et al. (2007), Rawson and Dunlosky (2012), and Rowland (2014). Except for Rowland (2014) and Kingston and Nash (2011), none of the aforementioned researchers conducted a meta‐analysis and therefore did not answer our primary research question. Rowland (2014) covered the psychological literature on the testing effect and did not focus on educational contexts. Kingston and Nash (2011) focused on formative assessment only and did not analyse the testing frequency.

#### 1.4.1 Potential contribution of this review

This review has the potential of providing an up‐to‐date, rigorous overview of the current research base regarding the effects of testing frequencies in primary and secondary education. Through systematic literature searches, quality appraisal of included studies and meta‐analytic procedures, we aim to present valid insights on both positive and adverse outcomes of different testing schemes, which may be of guidance to teachers, researchers and policy makers in search for answers on what constitutes an optimal testing frequency in various primary and secondary educational settings. Our primary outcome, students' academic achievement, should be highly relevant to all educational stakeholders. Our secondary outcome, testing anxiety, has not been meta‐analysed in the earlier related reviews (Adesope et al., 2017; Phelps, 2012; Yang et al., 2021) and, as anxiety is often regarded as the most important variable to understand the role of emotion for performance (von der Embse et al., 2017), it ought to be of broad interest to educational stakeholders.

## 2 OBJECTIVES

Our primary research question for this review is: *What are the effects of different testing frequencies on student achievement?*

Our secondary research question is: *What are the effects of different testing frequencies on measures of students' testing anxiety?*

We intend to further investigate our primary and secondary research questions by examining potential moderators, where we take a special interest in the moderating effects of test subject (reading/math/etc), grade level (kindergarten/1–3 grade/4–6 grade/etc.), type of test (formative/summative and high/low stakes), the duration of the intervention (short/long), and the gender composition of the sample. That is, our third research question is: *How are the effects of different testing frequencies on student achievement and testing anxiety moderated by subject, grade, type of test, duration of the intervention, and gender?*

## 3 METHODS

### 3.1 Criteria for considering studies for this review

#### 3.1.1 Types of studies

In this review, eligible study designs will be those applying a treatment‐control group design or a comparison design. Both randomised controlled trials (RCT) and quasi‐experimental studies (QES) are eligible for inclusion.

We will define a control group as ‘business as usual’, thus being exposed to their normal testing regimen. A comparison group design is defined as a design where all intervention groups are exposed to manipulated testing frequencies in the intervention period, and thus no groups are exposed to their normal testing regimens. Hence, this might also include groups exposed to no testing in the intervention period, given that this is not their usual testing regimen.

Furthermore, studies need to assign at least two ‘units’ (e.g., schools, classes, or students) to the treatment group and two units to the control group to be included. Treatment effects are difficult to separate from unit effects in studies with only one unit in either the treatment group or the control group.

#### 3.1.2 Types of participants

The eligible population includes students attending either primary or secondary school. In most countries, this means from kindergarten until grade 12. Students can be of any age, as long as they are enroled in one of these grades.

In some countries, kindergarten is not a part of the formal school system but rather a form of child care or pre‐school (e.g., in the United Kingdom). Therefore, we specify that kindergarten must be a grade within primary school to be included in this review. Tests of achievement in pre‐school child care settings are likely to be incomparable to those applied in a school setting. Furthermore, in countries where kindergarten constitutes a form of child care rather than a school setting, many children are likely not to attend, potentially making this population different from children in primary and secondary education. For example, in the United Kingdom as many as 29 percent of pre‐school aged children did not attend any formal child care (Department for Education, 2018).

We will exclude studies with participants enroled in tertiary education, such as universities. The student population found in higher education is fundamentally different to that found in K‐12, not only because of differences in age, but also because higher education is neither obligatory nor close to universal. Course structures, subjects, and curricula typically differ as well. For these reasons, we believe that pre‐school and tertiary settings are sufficiently different from primary and secondary school settings to justify a restriction of the review to the latter settings.

We will make no restrictions on the types of schools in which the interventions are performed, as long as the interventions are performed within a school setting. Studies carried out in both regular schools, boarding schools, special schools, and so forth, will be eligible for inclusion. We also include studies conducted in summer schools or after‐school programmes as long as these types of education are parts of the formal school system and the intervention has explicit academic aims.

We aim to include all types of students, not basing any eligibility criteria on students' background characteristics (such as socioeconomic status, or whether the students are at risk academically) or mental and physical capabilities.

#### 3.1.3 Types of interventions

We will include interventions that manipulate the frequency at which students are tested in a given period, and the number of tests given must differ between the intervention and comparison/control groups. Applied tests must be recorded on paper, computer, tablet, and so forth. Orally performed tests are included if their results are recorded.

If the intervention contains additional co‐interventions we will exclude the study. That is, if different testing regimens are combined with other co‐interventions (examples could be tutoring or peer‐assisted learning strategies), either in the control/comparison group or the treatment group or both, the intervention will not be eligible for inclusion. This is due to the possible confounding effect of an additional co‐intervention, which might drive the possible effects (or non‐effects) found for different testing regimens.

We will not restrict the eligibility of interventions on the basis of intervention length. Thus, we are interested in both interventions, which last for only a month, and interventions, which might last for several years. The effects of a testing regimen of four times a week used only for a month might be different from the effects found if the same testing regimen was implemented throughout the school year. We will record intervention length in our data extraction to allow for analytical categorisations and comparisons between interventions on the basis of intervention length.

Only interventions performed in a school setting are relevant for this review. Thus, experiments performed in laboratories will be excluded. This restriction is imposed because we are interested in the effects of different testing regimens in real school settings and educational systems. We will also include specific interventions, such as progress monitoring or curriculum‐based measurements, if the only components in these interventions are changes in testing frequency. If increases in testing frequencies are accompanied by other instructional or component changes, the interventions will not be eligible due to the risk of confounding effects.

#### 3.1.4 Types of outcome measures

We will include studies that test the effect of changing the testing frequency on either our primary outcome (measures of academic achievement), or our secondary outcome (measures of testing anxiety), or both. We describe which measures of academic achievement and testing anxiety that qualify below.

##### Primary outcomes

Our primary outcome of interest is academic achievement. In this review, we do not restrict measures of academic achievement to specific subjects, such as math or reading. Thus, measures of academic achievement in, for example, history classes or science classes will also be relevant for this review. The effect of the intervention must be tested using a test that is not identical to the one used during the intervention. Using identical tests may inflate effect sizes due to familiarity and recognition rather than actual learning (Adesope et al., 2017).

We aim to include a wide range of tests of academic achievement. In order for a test to be an eligible outcome, the answers must be recorded on paper, computer, tablet, and so forth. Orally performed tests are included if their results are recorded and administered by others than the students themselves.

We will include both formative and summative tests, as well as low stakes and high stakes tests. As the type of test may influence the effects, we intend to use the type of test in our moderator analyses (see Section 3.3.10). The definition of and separation between summative and formative tests, and high and low stakes tests, is not always clear cut. In ambiguous cases, we will, as far as possible, follow the study authors' definitions.

##### Secondary outcomes

As a secondary outcome measure, we will include tests of socio‐emotional outcomes and well‐being, if they relate specifically to the measurement of testing anxiety. Thus, a wide range of testing anxiety scales are eligible, examples being the Test Anxiety Inventory (TAI), the Test Anxiety Scale for Children (TASC), the Test Anxiety Inventory for Children and Adolescents (TAICA), the Test Anxiety Scale (TAS), the Children's Test Anxiety Scale (CTAS), and the Friedben Test Anxiety Scale (FTAS). However, measures of socio‐emotional outcomes related to testing anxiety can also be subtests from more general tests. An example of such a subtest is the anxiety content subscale from the Behaviour Assessment Scale for Children, Second edition (BASC‐2‐TA).

Also regarding testing anxiety, we are interested in both formative and summative tests, and low and high stakes tests. As summative tests and higher stakes may have different effects on anxiety compared to formative and lower stakes tests, we intend to examine whether these types of tests moderate effect sizes based on testing anxiety tests (see Section 3.3.10).

### 3.2 Search methods for identification of studies

#### 3.2.1 Electronic searches

We will conduct electronic searches in the following bibliographic databases:

- Academic Search Premier (EBSCO‐host)
- ERIC (EBSCO‐host)
- PsycInfo (EBSCO‐host)
- Socindex (EBSCO‐host)
- Teacher Reference Center (EBSCO‐host)
- EconLit (EBSCO‐host)
- Science Citation Index (Web of Science)
- Social Science Citation Index (Web of Science)
- Sociological Abstracts (ProQuest)

**Search terms**

An example of our search strategy from ERIC is shown below. A full description of modifications used in the searches of the specific databases will be added to the final review.

**Table:** 

**S12**	S3 AND S6 AND S9 AND S10 AND S11
**S11**	TI (achiev* OR learn* OR perform* OR improv* OR progress* OR result* OR impact* OR attain* OR success* OR ‘test* anxiety’ OR wellbeing) OR AB (achiev* OR learn* OR perform* OR improv* OR progress* OR result* OR impact* OR attain* OR success* OR ‘test* anxiety’ OR wellbeing)
**S10**	TI (student* OR pupil* OR child* OR adolescen* OR youth* OR young*) OR AB (student* OR pupil* OR child* OR adolescen* OR youth* OR young*)
**S9**	S7 OR S8
**S8**	AB (grade* N1 (1 OR 2 OR 3 OR 4 OR 5 OR 6 OR 7 OR 8 OR 9 OR 10 OR 11 OR 12)) OR AB (class* OR school* OR kindergarten* OR ‘primary education’ OR ‘secondary education')
**S7**	TI (grade* N1 (1 OR 2 OR 3 OR 4 OR 5 OR 6 OR 7 OR 8 OR 9 OR 10 OR 11 OR 12)) OR TI (class* OR school* OR kindergarten* OR ‘primary education’ OR ‘secondary education')
**S6**	S4 OR S5
**S5**	AB (‘test enhanced learning’ OR ‘testing effect*’ OR ‘testing phenomenon’ OR ‘frequency of testing’ OR ‘cumulative testing’ OR ‘progress monitoring’ OR 'curriculum‐based')
**S4**	AB (test* OR assess* OR measur* OR exam* OR quiz*) AND AB (frequen* OR repeat* OR interim* OR formative* OR summative* OR dail* OR week* OR month* OR annual* OR year*)
**S3**	S1 OR S2
**S2**	TI (‘test enhanced learning’ OR ‘testing effect*’ OR ‘testing phenomenon’ OR ‘frequency of testing’ OR ‘cumulative testing’ OR ‘progress monitoring’ OR 'curriculum‐based')
**S1**	TI (test* OR assess* OR measur* OR exam* OR quiz*) AND TI (frequen* OR repeat* OR interim* OR formative* OR summative* OR dail* OR week* OR month* OR annual* OR year*)

#### 3.2.2 Searching other resources

When relevant studies or reviews are identified, we will check the reference lists of these to check if additional relevant literature has been cited (citation‐tracking). For all relevant reviews we will also perform forward citation tracking. Furthermore, we will conduct forward and backward citation tracking on all included primary studies.

We will contact international experts to identify unpublished and ongoing studies, and provide them with the inclusion criteria for the review along with the list of included studies, asking for any other published, unpublished or ongoing studies relevant to the review. We will primarily contact corresponding authors of the related reviews mentioned in Section 1.4, but contacts will be extended to others if we find references to or mentions of ongoing studies in screened publications.

We will also conduct a hand search of the following journals, to make sure that all relevant articles are found. The following five journals are selected on the basis of our initial pilot search, where they included the most among relevant articles:

- *Assessment in Education: Principles, Policies and Practice*
- *Journal of Educational Research*
- *Educational Assessment*
- *Journal of Educational Psychology*
- *School Psychology Review*

The hand search will focus on editions published between 2018 and 2021 to secure recent unpublished articles, which have not yet been indexed in the bibliographic databases.

**Search for systematic reviews**

We will search for other relevant systematic reviews in the following resources:

- Campbell Systematic Reviews—https://campbellcollaboration.org/
- Cochrane Library—https://www.cochranelibrary.com/
- Centre for Reviews and Dissemination Databases—https://www.crd.york.ac.uk/CRDWeb/
- EPPI‐Centre Systematic Reviews—Database of Education Research—https://eppi.ioe.ac.uk/cms/Databases/tabid/185/Default.aspx

**Grey literature search**

We will search specifically after three types of grey literature: working papers, reports, and dissertations. Some of the bibliographic databases also cover grey literature (e.g., ERIC). We will search the following resources for grey literature:

- ProQuest Dissertations & Theses Global (dissertations) (EBSCO‐host)
- EBSCO Open Dissertations (dissertations) (EBSCO‐host)
- Open Grey (reports, working papers, dissertations)—http://www.opengrey.eu/
- Google Scholar (reports, working papers, dissertations)—https://scholar.google.com/
- Google searches (reports, working papers, dissertations)—https://www.google.com/
- Social Care Online (reports, working papers, dissertations, systematic reviews)—https://www.scie-socialcareonline.org.uk/
- Social Science Research Network (working papers)—https://www.ssrn.com/index.cfm/en/
- Mathematica (reports)—https://www.mathematica.org
- MDRC (reports, working papers)—https://www.mdrc.org/
- Abt Associates (reports) ‐ https://www.abtassociates.com/
- American Institutes for Research (reports)—https://www.air.org/
- WestEd (reports)—https://www.wested.org/
- WeStat (reports)—https://www.westat.com/
- SRI (reports)—https://www.sri.com/

Further resources for identifying grey literature may be added during the search process. A final list of grey literature resources will be included in the appendix of the review.

### 3.3 Data collection and analysis

#### 3.3.1 Selection of studies

The screening process for relevant studies will be divided into two stages: (1) screening on title and abstract, and (2) screening on full text. To ensure the quality of the screening process and reduce potential errors, we will make use of independent double screening at both stages (Polanin et al., 2019; Stoll et al., 2019). The screeners will be blind to each other's work until comparing final judgements. If the two screeners cannot agree on the inclusion/exclusion of a specific reference, the reference will be sent to one of the review authors for final judgement.

We will conduct a pilot‐screening for each screening stage and each screener. In the pilot screening of title and abstract, the review team will screen and compare 80–100 references. The review team will then discuss and resolve potential disagreements and uncertainties. If the interrater agreement is above 90% in the pilot, the screeners will continue to screen the rest of the references. If the interrater agreement is below 90% in the first pilot, the review team members will perform a second pilot screening to ensure reliability. At the full text stage of the screening process, the pilot will consist of 8–10 studies. The pilot procedure at second level is otherwise identical to the process described for first level. The review team will meet with regular intervals to discuss uncertainties and minimise ‘coders' drift’ (Polanin et al., 2019). The screening tool and guidance questions for screeners can be found in Supporting Information Appendix 1. Potential changes to the tool will be discussed during the pilot.

We will present the overall search and screening process in a flow chart in the final review.

During the screening process, none of the review authors or review team members will be blind to the authors, journals, or institutions responsible for the publication of eligible studies.

#### 3.3.2 Data extraction and management

Two members of the review team will independently extract and code data from the included studies. Before that, the coding tool will be piloted and potentially revised. See Supporting Information Appendix 2 for the current version of the tool. From all included studies, we extract data on publication characteristics, study characteristics, participant characteristics, intervention characteristics, control/comparison characteristics, and outcome characteristics. If any disagreement or uncertainty emerges during the data extraction process, a third reviewer with the appropriate expertise will be consulted.

All extracted data will be stored electronically using EPPI Reviewer 4 and Microsoft Excel.

#### 3.3.3 Assessment of risk of bias in included studies

Two members of the review team will independently assess the risk of bias for each study and the included study outcomes. The review team members will discuss disagreements in their ratings, and if necessary, a third review team member will be contacted for final agreement. We will report risk of bias assessments for all included effect sizes in the final review.

For included non‐randomised studies, we will assess the risk of bias for all included outcomes applying Cochranes ROBINS‐I tool (Sterne et al., 2016). For all included randomised studies, we will assess the risk of bias of all outcome measures using a revised version of Cochrane's risk of bias tool, ROB‐2 (Eldridge et al., 2016; Sterne et al., 2019). In this section, we briefly outline the characteristics of each tool.

##### ROBINS‐I

The ROBINS‐I tool covers seven domains. These seven domains broadly cover types of biases, which might be introduced in non‐randomised trials. The domains in ROBINS‐I are:

1. Confounding bias
2. Selection bias
3. Classification bias
4. Deviation bias
5. Missing data
6. Measurement bias
7. Reporting bias

In ROBINS‐I, every outcome in a study that is relevant for the review is rated on each domain as either having a ‘low’, ‘moderate’, ‘serious’, or ‘critical’ level of bias. In cases without sufficient evidence for rating the bias level, the outcome gets a rating of ‘no information'. If a study outcome receives a ‘critical’ rating on at least one domain, it is considered too biased to provide useful evidence on the effects of the intervention. As a consequence, the outcome is excluded from the data synthesis. We will not continue the risk of bias assessment of an outcome measure if a domain is rated ‘Critical'.

##### ROB‐2

The five domains in ROB‐2 cover types of biases potentially influencing the results found in RCTs. These are:

1. Bias arising from the randomisation process (pre‐intervention)
2. Bias arising from deviations from the intervention
3. Bias arising from missing outcome data
4. Bias arising from the measurement of outcomes
5. Bias arising from the selection of reported results

In each domain of the ROB‐2 tool, every outcome measure is rated as either calling for ‘low risk of bias’, ‘some concerns’, or ‘high risk of bias'.

Both tools have in common that an overall rating is made on the basis of the domain ratings. For example, a ‘Serious risk of bias’ in multiple domains of the ROBINS‐I assessment tool may lead to a decision of an overall judgement of ‘Critical’ risk of bias for that outcome, and it will be excluded from the data synthesis. Outcome measures which have been excluded due to multiple ratings of ‘Serious’ in individual domains will be listed in the final review, along with reasons for exclusion. The overall rating of the study also contains an assessment of the overall bias direction for the assessed outcomes in both tools. A further commonality is that both tools require pre‐specification of the effect type that will be assessed. We are most interested in, and believe that most studies will report estimates that are closer to, the effect of starting and adhering to the intervention than the effect of assignment to the intervention.

In the case of an RCT, where there is evidence that the randomisation has gone wrong or is no longer valid, we will assess the risk of bias of the outcome measures using ROBINS‐I instead of ROB‐2. Examples of reasons for assessing RCTs as non‐randomised studies may include studies showing large and systematic differences between treatment conditions while not explaining the randomisation procedure adequately; studies with large scale differential attrition between conditions in the sample used to estimate the effects; or studies selectively reporting results for some part of the sample or for only some of the measured outcomes. In such cases, differences between the treatment and control conditions are likely systematically related to other factors than the intervention and the random assignment is, on its own, unlikely to produce unbiased estimates of the intervention effects. Therefore, as ROBINS‐I allows for an assessment of for example confounding, we believe it is more appropriate to assess effect sizes from studies with a compromised randomisation using ROBINS‐I than ROB‐2. If so, we will report this decision as part of the risk of bias assessment of the outcome measure in question. As other effect sizes assessed with ROBINS‐I, these effect sizes may receive a ‘Critical’ rating and thus be excluded from the data synthesis.

##### Definition of critical confounders

ROBINS‐I dictates that reviewers should define critical confounders relevant to most or all eligible studies at the protocol stage. In the case of this review, we define the critical confounders as performance at baseline, and gender. Other important confounders may be for example the age and grade of the students, and students' socioeconomic status. If these are unbalanced between the treatment and control group, or the comparison groups, the lack of balance will be reflected in a higher rating (i.e., defining critical confounders does not imply that other confounders will not be considered). However, we anticipate that most studies will compare students of the same age and in the same grades, and differences in socioeconomic status will often be captured by performance at baseline.

Confounding happens when prognostic factors determine the allocation of participants into treatment conditions. Uncontrolled confounding will bring about systematic differences between the experimental conditions and thus compromises comparability. Confounding factors can be observable (e.g., age) or unobservable to the researcher (e.g., personal motivation). Both performance at baseline and gender are observable confounding factors. Inherently, unobservable confounding factors are harder for researchers to examine and control than observable confounding factors. Quasi‐experimental methods that explicitly try to avoid bias from unobservable confounding factors often use natural experiments to estimate effects, and include for example regression discontinuity designs and difference‐in‐differences.

Performance at baseline is generally considered a strong prognostic factor in relation to posttest outcomes (Hedges & Hedberg, 2007). Furthermore, frequent administration of tests might affect students who perform well at baseline differently than students who are struggling academically in terms of their motivation for learning. If high achievers continuously experience success in testing, this will most likely foster feelings of competence, confidence or relief. Opposed to this, if students struggling academically continuously experience failures when tested, it may foster feelings of incompetence, shame, and low self‐esteem (Russell & McAuley, 1986; Weiner, 2010).

Gender differences exist in relation to school performance (Holmlund & Sund, 2005). Thus, girls tend to outperform boys in reading, and boys usually outperform girls in mathematics (Stoet & Geary, 2013). Additionally, research findings indicate systematic gender differences in relation to testing anxiety. Girls are more likely to experience and report testing anxiety—a phenomenon that has been shown to affect academic achievement and life satisfaction negatively (DordiNejad et al., 2011; OECD, 2017; Segool et al., 2013; von der Embse et al., 2017). As previous research has highlighted the potential influences of gender in relation to school performance, and differential reactions to testing need not be captured by performance at baseline, we included gender as a critical confounder.

In each of the risk of bias assessments of outcome measures, we examine how the study authors have considered the two predefined critical confounding factors, either at the design stage or in the analysis.

There is a plethora of different designs (e.g., matching) and analytic methods (e.g., regression analyses) with which researchers can seek to adjust for the confounding of intervention effects. However, the appropriateness of such designs and measures depends on the specific characteristics of each study. That is, matching (as an example) is neither inherently ‘appropriate’ nor ‘inappropriate'. Thus, as part of the risk of bias analyses, we will conduct individual assessments of the appropriateness of methods and designs applied to counter biases.

#### 3.3.4 Measures of treatment effect

We expect that almost all studies found in this literature use continuous outcome measures. For continuous data, we will calculate the standardised mean difference (SMD) where possible, as our outcomes (academic achievement and testing anxiety) are measured and reported with a wide range of different scales. To correct for upward bias in small samples, we will use the small sample bias‐corrected Hedges' g in our analysis (Borenstein et al., 2009; Hedges, 1981; Lipsey & Wilson, 2001). Hedges' g and its standard error are calculated as (Lipsey & Wilson, 2001, pp. 47–49):

(1) $$ES_{g}=\left(1-\left(3/\left(4N-9\right)\right)\right)\times \left(\left(X_{1}-X_{2}\right)/S_{p}\right),$$

(2) $$SE_{g}=\sqrt{\left(\left(N/\left(n_{1}\times n_{2}\right)\right)+\left(ES_{g}^{2}/\left(2N\right)\right)\right)},$$

where *N* = *n*_1_ + *n*_2_ is the total sample size, *X_1_* and *X_2_* are the means in each group, and *S*_*p*_ is the pooled standard deviation defined as

(3) $$S_{p}=\sqrt{\left(\left(\left(n_{1}-1\right)\times S_{1}^{2}+\left(n_{2}-1\right)\times S_{2}^{2}\right)/\left(\left(n_{1}-1\right)+\left(n_{2}-1\right)\right)\right).}$$

Here, *s*_1_ and *s*_2_ denote the unadjusted standard deviations of the treatment and control groups. We will record both post‐ and pretest standard deviations if available. For our main analysis, we will calculate SMD's with posttest standard deviations, as these values are more likely to be reported and due to the possibility of floor effects at pretest. Only when posttest standard deviations are not available, but pretests standard deviations are, we intend to use these as replacements for possible missing values. However, we do recognise that using pre‐test standard deviations can be advantageous, due to the possibility of treatment affecting variability (Lipsey & Wilson, 2001). If we detect a difference between pre and posttest standard deviations, we will thus check the sensitivity of our calculated SMD's—for further details see Section 3.3.11. We will use covariate adjusted means whenever available.

If some studies report intention‐to‐treat (ITT) estimates of the mean difference and others treatment‐on‐the‐treated (TOT) or local average treatment effects (LATE), we will test the sensitivity to including these different estimates. If there is a mix of studies with some reporting change scores and others reporting final values, we will contact the trial investigators and request the final values. If these are unobtainable, we will analyse change scores and final values separately.

If included studies report dichotomous outcome data, we will use the methods described in Sánchez‐Meca et al. (2003) to transform the outcome data into SMDs.

#### 3.3.5 Unit of analysis issues

Errors in statistical analysis can occur when the unit of allocation differs from the unit of analysis. In cluster‐randomised trials, participants are randomised to treatment and control groups in clusters, either when data from multiple participants in a setting are included (creating a cluster within the school or community setting), or when participants are randomised by treatment locality or school. QES may also include clustered assignment of treatment. Effect sizes and standard errors from such studies may be biased if the unit of analysis is the individual and an appropriate cluster adjustment is not used (Higgins & Green, 2011). If possible, we will adjust effect sizes individually using the methods suggested by Hedges (2007) and information about the intra‐cluster correlation coefficient (ICC), realised cluster sizes, and/or estimates of the within and between variances of clusters. If it is not possible to obtain this information consistently across included studies, we will adjust the effect sizes using estimates from the literature of the ICC in Hedges and Hedberg (2007), and assume equal cluster sizes. We will use an ICC of 0.11, which approximately corresponds to the average of ICCs taken over grades, maths and reading tests reported in tab. 2 and 3 in Hedges and Hedberg (2007, pp. 68–69). We will test if our results are sensitive to this choice by using ICCs of 0 (the theoretical lower bound) and 0.32 (the empirical upper bound in the same two tables). To calculate an average cluster size, we will divide the total sample size in a study by the number of clusters (typically the number of classrooms or schools).

Studies including multiple interventions per individual may also be included, but only one intervention group will be coded and compared to the control or comparison group to avoid overlapping samples. In some cases, several studies may have used the same sample of data, for example, studies using the same administrative data. We will review all such studies, but will only include one estimate of the effect from each sample of data in the meta‐analysis to avoid dependencies. The choice of which estimates to include will be based on our risk of bias assessments of the estimates. We will choose the estimates that we judge to have the least risk of bias.

#### 3.3.6 Dealing with missing data

Studies must permit calculation of a numeric effect size for the outcomes to be eligible for inclusion in the meta‐analysis. Where studies have missing summary data, such as missing standard deviations or means, we will derive these where possible from, for example, *F*‐ratios, *t*‐values, *χ* ^2^ values and correlation coefficients using the methods suggested by Lipsey and Wilson (2001). If these statistics are also missing, the review authors will request information from the study investigators. If missing summary data necessary for the calculation of effect sizes cannot be derived or retrieved, the study results will be reported in as much detail as possible, i.e., the study will be included in the review but excluded from the meta‐analysis. Missing data and attrition rates for the individual studies are assessed with the risk of bias tools, where both ROB‐2 and ROBINS‐I have specific domains focusing on biases arising from missing data (Sterne et al., 2016, Sterne et al., 2019).

#### 3.3.7 Assessment of heterogeneity

Heterogeneity can stem from either an expected variation in effects or from sampling errors in included studies. In this review, we assume that variation in effects will occur and will therefore use a random effects model in our main analysis (see also Section 3.3.9). Consequently, we expect to find heterogeneity in our analyses. We aim to assess the level of heterogeneity with the Q‐statistic, the I‐squared, and τ‐squared statistics (Higgins et al., 2003), as well as prediction intervals (defined below).

We will report prediction intervals to examine and show how effects are dispersed. Prediction intervals are based on the mean effect size and the standard deviation of effect sizes, instead of standard errors, which are used in the calculation of confidence intervals. We will calculate prediction intervals wherein effects will lie 95% of the time. Since the mean and the standard deviation can only be estimated with some error, we calculate the lower and upper limits of the prediction intervals with the modifications provided in formulas 4 and 5 (Borenstein et al., 2017; Higgins et al., 2009):

(4) $$LL_{pred}=X-t_{df}\times \sqrt{\left(\tau^{2}+V_{X}\right)},$$

(5) $$UL_{pred}=X+t_{df}\times \sqrt{\left(\tau^{2}+V_{X}\right)},$$

where *X* is the estimated mean effect size, *t*_*df*_ is the critical *t*‐value for our degrees of freedom, *τ* ^2^ is the estimated between‐study variance, and *V*_*X*_ is the variance of the mean effect size.

#### 3.3.8 Assessment of reporting biases

Reporting bias might refer to both publication bias and selective reporting of outcome data and results. We will assess bias from selective reporting of outcome data and results in both ROB‐2 and ROBINS‐I.

As different methods may yield different results, we intend to use different methods to assess the extent of publication bias. First, we will show funnel plots and examine whether they are asymmetric (Higgins & Green, 2011). To formally test for asymmetry, we will use a version of Egger's test (Egger et al., 1997) suggested by Rodgers and Pustejovsky (2020). Egger's test examines asymmetry by including a measure of effect size precision—often, as in our case, the standard error—as a predictor in a meta‐regression with effect sizes as the outcome variable. A significant coefficient on the standard error is interpreted as evidence of asymmetry. However, Pustejovsky and Rodgers (2019) showed that the original Egger's test often rejects the null hypothesis of no asymmetry at higher rates than the chosen level of statistical significance (i.e., the Type I errors were inflated). Rodgers and Pustejovsky (2020) tested a version of Egger's test, which handled effect size dependence within studies by using robust variance estimation (RVE). In their simulations, this ‘Egger Sandwich’ test had better properties in terms of Type I errors than the original Egger's test, and other tested methods. As Rodgers and Pustejovsky (2020), we will interpret the rejection of the null hypothesis of no asymmetry in a one‐sided test with significance level 0.05 as an indication of asymmetry.

It is important to note that asymmetric funnel plots are not necessarily caused by publication bias (and publication bias does not necessarily cause asymmetry in a funnel plot). If asymmetry is present, we will consider possible reasons for the asymmetry and test how sensitive our results are to publication bias using the method developed by Mathur and VanderWeele (2020). Furthermore, the Egger Sandwich test may have limited capacity to detect publication bias when the number of included studies is small (Rodgers & Pustejovsky, 2020), which may be the case in our review. As a sensitivity analysis, we will use selection models that may identify and correct for the presence of publication bias (e.g., Andrews & Kasy, 2019; Hedges & Vevea, 2005; Hedges,^1992^). The selection model tested in Rodgers and Pustejovsky (2020) showed some signs of an inflated Type I error rate but had better statistical power of detecting bias than all versions of Egger's test unless the probability of censoring of nonsignificant results was very large. We therefore believe that the two methods have complementary strengths. We describe our use of selection models in more detail in see Section 3.3.11.

#### 3.3.9 Data synthesis

The data synthesis will be conducted in the following steps: First, we will provide descriptive summaries of the contextual, methodological, and outcome characteristics for the studies included in the data synthesis. We intend to perform all statistical analyses in R.

Our main effects analysis will be conducted first. Along with the main analysis, we will present forest plots, prediction intervals, and heterogeneity statistics. Second, as far as our data permit, we will conduct our proposed moderator and sensitivity analyses (described in the next sections). The main effects analysis will compare the high frequency test conditions with the low frequency test conditions of interventions. We assume a random‐effects model. We will use inverse‐variance weighted mean effect sizes for all parts of the analysis and include effect sizes from both treatment‐control and treatment‐comparison studies. To estimate the overall effect size and heterogeneity parameters, we will use the RVE methods developed by Pustejovsky and Tipton (2021). Their method is implemented in three steps.

First, we identify an appropriate working model based on the features of our sample. Important features to consider is whether there are dependencies between effect sizes that arise because the same sample is tested on different tests (‘correlated effects’) and because different samples are included in the same study (‘hierarchical effects’). As both these types of dependencies are conceivable in our case, this feature is an advantage over the original RVE method developed by Hedges, Tipton, and Johnson (2010). As in the original RVE method, a baseline value for the correlation between pairs of effect sizes from the same study (*ρ*) has to be specified. We will choose 0.6, as suggested by Pustejovsky and Tipton (2021), but test if our results are sensitive to lower (0.4) and higher (0.9) values. We chose the latter value because some of the results in Pustejovsky and Tipton (2021) were sensitive to using values of *ρ* higher than 0.8.

Second, based on the chosen working model, we will estimate a meta‐regression using a combination of the *clubSandwich* (Pustejovsky, 2020) and *metafor* (Viechtbauer, 2010) packages in R. We will use the *clubSandwich* package to specify the correlation structure between effect size estimates within studies. Then, we will estimate the random effects variance components, including *τ* ^2^, inverse‐variance weight matrices, and the meta‐regression coefficients using the restricted maximum likelihood (REML) procedure in the *metafor* package.

Third, we will calculate confidence intervals based on the RVE standard errors obtained from the *clubSandwich* package. These standard errors are adjusted for small‐sample bias as suggested by Tipton (2015) and Tipton and Pustejovsky (2015). We intend to report 95% confidence intervals for all analyses.

Our primary outcome variable is effect sizes based on measures of academic achievement and our secondary outcome variable is effect sizes based on measures of testing anxiety. Besides separating between academic achievement and testing anxiety, we intend to include all types of tests and subjects in each category in the main effects analysis and examine heterogeneity between types and subjects in the moderator analysis. Corresponding to our first two research questions, the meta‐regression model will include indicators for how the testing frequency of the treatment group differs from the baseline provided by the control/comparison group. In a best case scenario, we intend to include indicators for 0 versus more tests, 1 test versus more tests, 2 tests versus more tests, and so forth. However, as data might be limited, we realise that this thorough examination of specific testing frequencies might be rendered impossible by the number of studies. If so, we will coarsen the categories by applying fewer and broader indicators until we can estimate the coefficients without ending up with adjusted Sattertwaithe degrees of freedom below 4 for any coefficient. We chose this threshold because Tipton (2015) suggested that RVE standard errors are unreliable when the degrees of freedom are below 4.

#### 3.3.10 Subgroup analysis and investigation of heterogeneity

To answer our third research question, we intend to conduct a moderator analysis to identify the characteristics that are possibly associated with smaller and larger effects on the primary and secondary outcomes. For the moderator analysis, we will use a similar meta‐regression method as for the main effects analysis. We intend to add all moderators in one regression, thus reducing the risk of misleading results due to correlated independent variables. We will start by pooling all effect sizes and include the following types of moderators (described in Section 1.3):

1. Test subject (reading/math/etc)
2. Grade level (kindergarten/1‐3 grade/4‐6 grade/etc)
3. Type of test (formative/summative and high/low stakes)
4. Duration of intervention (measured in weeks)
5. Gender

We will report 95% confidence intervals for all regression parameters. The exact definition of moderators may be subject to change during the data extraction process. However, a preliminary version of the codebook, including more details on some of the moderators can be found in Supporting Information Appendix 2.

It is perhaps likely that we will not be able to include all moderators in one meta‐regression without running into problems with having too few (i.e., below 4) adjusted degrees of freedom. In that case, we will reduce the number of estimated coefficients in the following way.

First, we will prioritise moderators without missing observations.

Second, we will combine adjacent categories of testing frequencies that were not significantly different from one another in the main effects analysis into one category, and then re‐iterate. For example, if the main effects model contained four indicators, 0 versus more tests, 1 versus more tests, 2 versus more tests, and 3 versus more tests, and none is significantly different from the others, we will run a new model and test if the indicator for the combined first two categories is significantly different from the indicator for the combined last two categories. If these two indicators are significantly different from one another, we include one of them and use the other as the reference category.

Third, we will prioritise moderators in the order mentioned above. If we have to prioritise between moderators in this way, we will also report exploratory analyses that add single moderators to the main effects specification.

#### 3.3.11 Sensitivity analysis

To explore the sensitivity of our results, we intend to perform the following sensitivity analyses: Examination of distribution of effect sizes, examination of our effect size measurements, examination of methodological quality, and publication bias.

1) *Distribution of effect sizes*
  We will examine the distributions of effect sizes for each outcome category for the presence of outliers. If outliers are found, we will examine the sensitivity of the results by winsorising the outliers to the nearest non‐outlier value (Lipsey & Wilson, 2001).
2) *Effect size measurement*
  As stated in the main analysis section, we intend to estimate SMDs with posttest standard deviations as these values are more likely to be reported. If we find that differences exist, we will then check the sensitivity of our results by calculating alternative SMDs where the pretest standard deviations are used as default for the calculation of SMDs.
  We will also examine whether or not possible differences in baseline differences between the treatment and control group affects our results. If we find that such differences exist, we will calculate alternative SMDs taking into account these baseline differences.
3) *Methodological quality*
  To examine methodological quality, we will consider sensitivity analyses for each domain in the risk of bias assessments. The studies which have received a rating of either ‘high’ or ‘serious’ in a domain will be removed from the model to test for sensitivity of our results.
4) *Publication bias*
  To test for sensitivity regarding publication bias we will present a funnel plot and Egger's test (as described in Section 3.3.8). Furthermore, we intend to examine publication bias with selection models. Our procedure for this part of the analysis is described below:

Selection models typically use a weight function to represent the process of selection of studies (e.g., Hedges & Vevea, 2005). If we find enough studies, we will use the non‐parametric approach suggested by Andrews and Kasy (2019), which avoids making assumptions on the functional form of the publication probability as well as on the distribution of true effects. It is however more likely that the number of studies is too low for the non‐parametric approach to work well. In this case, we will either use Andrews and Kasy's generalised method of moments (GMM) approach or a fully parametric approach using maximum likelihood for estimation. The GMM approach requires fewer studies to work well than the non‐parametric approach and assumes a functional form for the publication probability, but makes no assumptions on the distribution of true effects. A parametric approach likely requires fewer studies to work well than the GMM approach, but assumes both a functional form and a normal distribution for the true effects (the model implemented by Andrews & Kasy, 2019, is similar to, for example, the ones that can be implemented using the R package *weightr* developed by Coburn & Vevea, 2019).

To implement the GMM or the parametric approach, we need to specify cutoffs or intervals, where the probability of publication may change. To avoid author bias, we define the cutoffs in advance. Our review will include both studies published in journals and ‘unpublished’ studies, such as government reports and working papers. Thus, the selection process which we will try to model represents the decision of authors to make their studies available in such a way that we will find them, rather than the decision of editors and reviewers to publish studies in scientific journals. In the absence of established guidelines for choosing cutoffs, we will use the cutoffs used by Andrews and Kasy (2019) in their empirical application that included unpublished studies. Here we define the *z*‐statistic *Z* = *ES*_*g*_/*SE*_*g*_ and let the probability of publication be denoted as *p*(*Z*). We then first normalise by setting *p*(*Z*) = 1 for *Z* ≥ 1.96. That is, the estimated probabilities of publication are defined in relation to the publication probability of studies with positive *Z* values corresponding p‐values at or below the conventional level of statistical significance *p* ≤ 0.05 (see e.g., Andrews & Kasy, 2019, for the necessity of normalising). We then allow for the probability of publication to depend on the sign of the *z*‐statistic *Z* and use the following cutoffs: *Z* < −1.96; −1.96 ≤ *Z* < 0; 0 ≤ *Z* < 1.96.

Should the number of studies be too low to obtain precisely estimated probabilities with this number of cutoffs, even with the parametric approach, we will use one single cutoff, where the absolute value of *Z* < 1.96 and thus the normalisation *p*(|*Z*| > 1.96) = 1. Once we have obtained estimates of *p*(*Z*), we can use them to calculate the median unbiased estimator proposed by Andrews and Kasy (2019).

However, our effect size estimates, *ES*_*g*_, and corresponding standard errors, *SE*_*g*_, will not necessarily stem from the estimates used by authors, editors, and reviewers to decide whether a paper should be published. For example, we will use the raw standard deviations to calculate effect sizes, not model‐based standard errors, *t*‐statistics, or *p*‐values, which may be more likely input to the publication decision. Furthermore, all selection models, also non‐parametric ones, make some relatively strong assumptions about how the selection process generates publications. For example, Andrews and Kasy's (2019) non‐parametric approach assumes that studies with smaller standard errors do not have systematically different effect estimates. This assumption of independence between standard errors and effect sizes may not hold if larger studies for instance recruit schools/students that have systematically different characteristics. Although McShane et al. (2016) find that selection models perform better than alternative approaches (p‐curve and p‐uniform approaches), they express doubts about using selection models to obtain a single bias‐corrected estimate of the overall effect. They discuss several reasons why the results of selection models may be problematic: (a) the publication decision is likely based on more than just statistical significance, (b) it is difficult to know which effect estimate(s) the decision is based on, or (c) the effects across studies of similar interventions may not be independent. For all these reasons, we will interpret the corrected effect size estimates with caution.

## CONTRIBUTIONS OF AUTHORS

The lead author of the review is Morten Kjær Thomsen. The co‐authors of the review are Julie Kaas Seerup, Jens Dietrichson, Anja Bondebjerg, and Bjørn Christian Arleth Viinholt. The responsibilities of the authors are given below.

- Content: Morten K. Thomsen, Julie K. Seerup, Jens Dietrichson, and Anja Bondebjerg.
- Systematic review methods: Julie K. Seerup, Jens Dietrichson, and Anja Bondebjerg.
- Statistical analysis: Julie K. Seerup and Jens Dietrichson.
- Information retrieval: Bjørn C. A. Viinholt.

## CONFLICT OF INTERESTS

The authors declare that there are no conflict of interests.

## SOURCES OF SUPPORT

### Internal sources

- VIVE Campbell, Denmark

## Supporting information

Supporting information.

Supporting information.
