# Supplementary material for: PROTOCOL: Testing frequency and student achievement: A systematic review
Source: Campbell Syst Rev. 2022 Jan 7;18(1):e1212. doi: 10.1002/cl2.1212 (PMC8742135; doi:10.1002/cl2.1212)
Supplement: Supplementary file 1 — Supporting information. [file CL2-18-e1212-s002.pdf]

Appendix 1. Criteria for screening

This appendix contains the guidance questions for the screening tool. Please note that changes might be made to the tool during pilot screening. The screening process is double blinded in both title/abstract and in full text screening. If screening disagreements cannot be resolved by the two screeners, one of the review authors will act as a third screener.

First level screening on title and abstract

Table A1: Screening tool for title and abstract screening

Include	Answer “yes” or “uncertain” to all guidance questions 1-5
Exclude	Answer “no” to at least one of the guidance questions 1-5

Question 1: Is the abstract written in Danish, English, German, Norwegian or Swedish?

- Yes: Include
- Uncertain: Include
- No: Stop screening and exclude

Guidance: Only studies providing abstracts in languages that at least two of the research members can understand are eligible for inclusion. In the current review, these languages are English, Danish, German, Norwegian, and Swedish.

Question 2: Are the participants in the interventions attending either primary or secondary school?

- Yes: Include
- Uncertain: Include
- No: Stop and exclude

Guidance: The population eligible for this review are students attending either primary or secondary school, which in most countries will mean from kindergarten until grade 12. In some countries, kindergarten class is not a part of primary school, but a form of preschool (e.g., the UK). Kindergarten must be considered a formal (first) year of primary school in order to be included in this review, meaning that interventions performed in preschool settings will be excluded.

Terminology of primary and secondary school

Within the English speaking world, there are three widely used systems to describe the age of the child: The American model, the English model, and the 'equivalent ages' of the student. This terminology extends into the research literature. See table A1 for a convenient comparison between the three models.

Table A2: Comparison between school models (grades, forms and ages)

Equivalent ages	US grades	US nicknames	England grades	England forms
4-5 years old	Pre-K	-	R	Reception
5-6 years old	K	-	1	Infants
6-7 years old	1	-	2	Top infants
7-8 years old	2	-	3	Junior 1
8-9 years old	3	-	4	Junior 2

9-10 years old	4	-	5	Junior 3
10-11 years old	5	-	6	Junior 4
11-12 years old	6	-	7	First
12-13 years old	7	-	8	Second
13-14 years old	8	-	9	Third
14-15 years old	9	Freshman	10	Fourth
15-16 years old	10	Sophomore	11	Fifth
16-17 years old	11	Junior	12	Lower sixth
17-18 years old	12	Senior	13	Upper sixth

Question 3: Is the study about administering tests with different intervals?

- Yes: Include
- Uncertain: Include
- No: Stop screening and exclude

Guidance: We are interested in studies that explore the effects of different testing regimens. Interventions using curriculum-based measurement progress monitoring may be eligible for inclusion, if they are the only methods used in the intervention (often, these methods are accompanied by other changes to the curriculum).

Question 4: Does the study report quantitative measures?

- Yes: Include
- Uncertain: Include
- No: Stop and exclude

Guidance: This review does not include qualitative research. If studies explicitly state that they only use qualitative methods, then exclude.

Second level screening on full text

Table A3: Full text screening tool

Include	Answer "yes" or "uncertain" to all guidance questions
Exclude: Wrong language	Answer "no" to question 5 (or to question 1 in T/A)
Exclude: Wrong intervention focus	Answer "no" to question 6 (or to question 2 in T/A)
Exclude: Wrong setting	Answer "no" to question 7 (or to question 3 in T/A)
Exclude: Wrong type of tests	Answer "no" to question 8
Exclude: Wrong study design	Answer "no" to question 9 (or to question 4 in T/A)

Note 1: A study might be eligible for exclusion in several domains.
Note 2: Studies included with an "uncertain"-judgement, should be sent to one of the review authors for assessment of eligibility.

Question 5: Is the full text written in Danish, English, German, Norwegian or Swedish?

- Yes: Include

- Uncertain: Include, and send to one of the review authors
- No: Stop screening and exclude

Guidance: Studies might provide abstracts in English, while the full text is written in another language. Only studies providing full texts in languages that at least two of the research members understand are eligible for inclusion. In the current review, eligible languages are English, Danish, Swedish, and Norwegian.

Question 6: Are the outcome measures focused on academic achievement?

- Yes: Include
- Uncertain: Include, and send to one of the review authors
- No: Stop and exclude

Guidance: The intervention must report results that can be used to examine academic achievement. Academic achievement need not be the sole purpose of the study.

Question 7: Does the intervention take place in a school setting?

- Yes: Include
- Uncertain: Include, and send to one of the review authors
- No: Stop and exclude

Guidance: Interventions must be implemented within a school setting. Laboratory-based interventions are excluded. School setting also includes summer schools and after-school programs. We will make no restrictions on types of schools in which the interventions are performed, thus both regular schools, boarding schools, and special schools will be eligible for inclusion.

Question 7: Does the intervention use written and non-identical tests to measure academic achievement or testing anxiety?

- Yes: Include
- Uncertain: Include, and send to one of the review authors
- No: Stop and exclude

Guidance: Both standardised and non-standardised tests as well as high-stakes exams and low-stakes practice tests will be included. The applied tests must be written down and answered on paper, computer, tablet etc. Interventions consisting of orally performed tests or self-quizzing are not relevant to this review (see Methods section). Measures and outcomes of academic achievement should not be based on repeated administration of identical tests. If tests are identical, chances are that students might learn the specific questions and answers by heart, thus introducing a confounding element in the identification of effects.

Question 8: Is the study an RCT or QES with more than one unit in treatment/control conditions?

- Yes: Include
- Uncertain: Include, and send to one of the review authors
- No: Stop and exclude

Guidance: We are interested in randomised controlled trials (RCT) or quasi-experimental studies (QES), which use a control/comparison design to examine effects. Such studies can have many labels, so it is important to read the study design section in order to make a qualified assessment. The most common sub-categories of randomised controlled trials and quasi-experimental studies can be found in table A4 (please note that this is not a complete list).

Table A4: Most common study designs

**Randomised controlled trials (RCTs):**	**Quasi-experimental studies (QES):**

Participants are allocated at random to control and treatment groups.	Participants are not allocated at random to control and treatment groups.
Individual randomised assignment:<br>Individuals are assigned to treatment and<br>control conditions at random (e.g., by coin toss or random number generator).<br><br>Cluster randomised assignment:<br>Groups are allocated to treatment and control conditions, e.g. classes or schools.<br><br>Stratified/blocked random assignment:<br>The sample is divided into stratas/blocks (e.g., grade levels), and then randomly<br>assigned to treatment and control conditions within these stratas/blocks.	Pseudo-randomisation:<br>Non-random sequences used for assignment to treatment/control<br>conditions (e.g., birth date, case number, alphabetic order etc.).<br><br>Matching:<br>Individuals are matched in pairs on observed characteristics (e.g., pre-test scores or age).<br><br>Other possible designs:<br>Cohort studies, difference-in-differences, regression-discontinuity designs, instrumental variable designs, case-control studies.
