# Supplementary material for: PROTOCOL: Testing frequency and student achievement: A systematic review
Source: Campbell Syst Rev. 2022 Jan 7;18(1):e1212. doi: 10.1002/cl2.1212 (PMC8742135; doi:10.1002/cl2.1212)
Supplement: Supplementary file 2 — Supporting information. [file CL2-18-e1212-s001.pdf]

# Appendix 2. Code book

**1 Publication Characteristics**
Authors
Publishing status (in press, published)
Publication year
Type of publication (journal article, dissertation, book)

**2 Study Characteristics**
Study location (country)
Study design (QES, RCT)
Number of sites included in study (districts, schools, classrooms)
Type of school (regular school, boarding school, special school)
Implementation quality (implementation problems, type of implementation problems)

**3 Participant characteristics**
Gender
Age
Grade
Background (SES, ethnicity, potential diagnoses)

**4 Intervention characteristics**
Overall testing regimen (short description)
Type of test (formative/summative; low stakes/high stakes; standardised/teacher developed)
Feedback component (yes/no)
Intervention length (duration in weeks)
Number of tests (total in intervention)
Test length (in minutes per test)
Interval between administrations of tests (in weeks)
Content area

**5 Control / comparison characteristics**
No test control (yes/no)
If test control: type of test, feedback component, number of tests, test duration, interval between administrations of tests, and content area

**6 Outcome characteristics (*repeat for all outcomes*)**
Sample size (for both treatment and control conditions)
Measurement timing
Content area
Type of test
Outcome (effect size measure/continuous outcome/dichotomous outcome/other)
